# Serum levels of fibrogenesis biomarkers reveal distinct endotypes predictive of response to weight loss in advanced nonalcoholic fatty liver disease

**DOI:** 10.1097/HC9.0000000000000254

**Published:** 2023-09-27

**Authors:** Morten A. Karsdal, Kate Hallsworth, Jadine Scragg, Diana J. Leeming, Ida F. Villesen, Leah Avery, Laura Haigh, Olivier Govaere, Sarah Wichmann, Guy Taylor, Sophie Cassidy, Stuart McPherson, Quentin M. Anstee

**Affiliations:** 1 Nordic Bioscience Biomarkers and Research A/S, Herlev, Denmark; 2 Translational and Clinical Research Institute, Faculty of Medical Sciences, Newcastle University, Newcastle upon Tyne, UK; 3 Newcastle NIHR Biomedical Research Centre, Newcastle upon Tyne Hospitals NHS Foundation Trust, Newcastle upon Tyne, UK; 4 Population Health Sciences Institute, Faculty of Medical Sciences, Newcastle University, Newcastle upon Tyne, UK; 5 Nuffield Department of Primary Care Health Sciences, University of Oxford, Oxford, UK

## Abstract

**Background:**

NAFLD is associated with activation of fibroblasts and hepatic fibrosis. Substantial patient heterogeneity exists, so it remains challenging to risk-stratify patients. We hypothesized that the amount of fibroblast activity, as assessed by circulating biomarkers of collagen formation, can define a “high-risk, high-fibrogenesis” patient endotype that exhibits greater fibroblast activity and potentially more progressive disease, and this endotype may be more amendable to dietary intervention.

**Methods:**

Patients with clinically confirmed advanced NAFLD were prescribed a very low-calorie diet (VLCD) intervention (∼800 kcal/d) to induce weight loss, achieved using total diet replacement. Serum markers of type III (PRO-C3) and IV collagen (PRO-C4) fibrogenesis were assessed at baseline every second week until the end of the VLCD, and 4 weeks post-VLCD and at 9 months follow-up.

**Results:**

Twenty-six subjects had a mean weight loss of 9.7% with VLCD. This was associated with significant improvements in liver biochemistry. When stratified by baseline PRO-C3 and PRO-C4 into distinct fibrosis endotypes, these predicted substantial differences in collagen fibrogenesis marker dynamics in response to VLCD. Patients in the high activity group (PRO-C3 >11.4 ng/mL and/or PRO-C4 >236.5 ng/mL) exhibited a marked reduction of collagen fibrogenesis, ranging from a 40%–55% decrease in PRO-C3 and PRO-C4, while fibrogenesis remained unchanged in the low activity group. The biochemical response to weight loss was substantially greater in patients a priori exhibiting a high fibroblast activity endotype in contrast to patients with low activity.

**Conclusions:**

Thus, the likelihood of treatment response may be predicted at baseline by quantification of fibrogenesis biomarkers.

## INTRODUCTION

Advanced NAFLD is associated with development of hepatic fibrosis, decreased liver function, and a high mortality rate due both to hepatic dysfunction and development of comorbidities related to cardiovascular disease, cancer, and kidney disease.^1,2^ NAFLD is characterized by substantial interpatient variation in disease severity and outcome.^3^ In the absence of pharmacological therapies, behavioral change (to initiate weight loss) is widely considered the mainstay of NAFLD therapy. However, it is notable that the therapeutic benefit of weight loss is inconsistent with many patients failing to achieve improvements in hepatic fibrosis even after sustained weight reduction.^4,5^ Targetable patient endotypes are gaining traction in fields such as cancer and osteoarthritis but are still lacking for NAFLD. While an improved understanding of the genetic modifiers of liver disease goes some way to address this,^6,7^ there remains a pressing medical need to better understand patient heterogeneity and identify predictive endotypes and so triage patients toward more personalized therapeutic strategies.^8^

To demonstrate the efficacy of antifibrogenesis treatments, the actual presence of ongoing fibroblast activity rather than a crude baseline fibrosis stage is critical. Supporting this assertion, several recent drug trials with PPARs and FGF-21^9–11^ that failed to attain their primary efficacy endpoints have, in *post hoc* analyses, consistently shown increased efficacy among patients with high baseline fibroblast activity.^9^ Patients with increased fibroblast activity have also been shown to have worse outcomes.^12^ Preferential targeting of this “high-risk, high-fibrogenesis” endotype is presently not implemented during recruitment into NAFLD clinical studies^9^ but is routinely adopted in other disease areas such as osteoporosis, where biomarkers of bone formation/resorption were pivotal for understanding patient heterogeneity and for successful drug development.^13^

**FIGURE 1:**
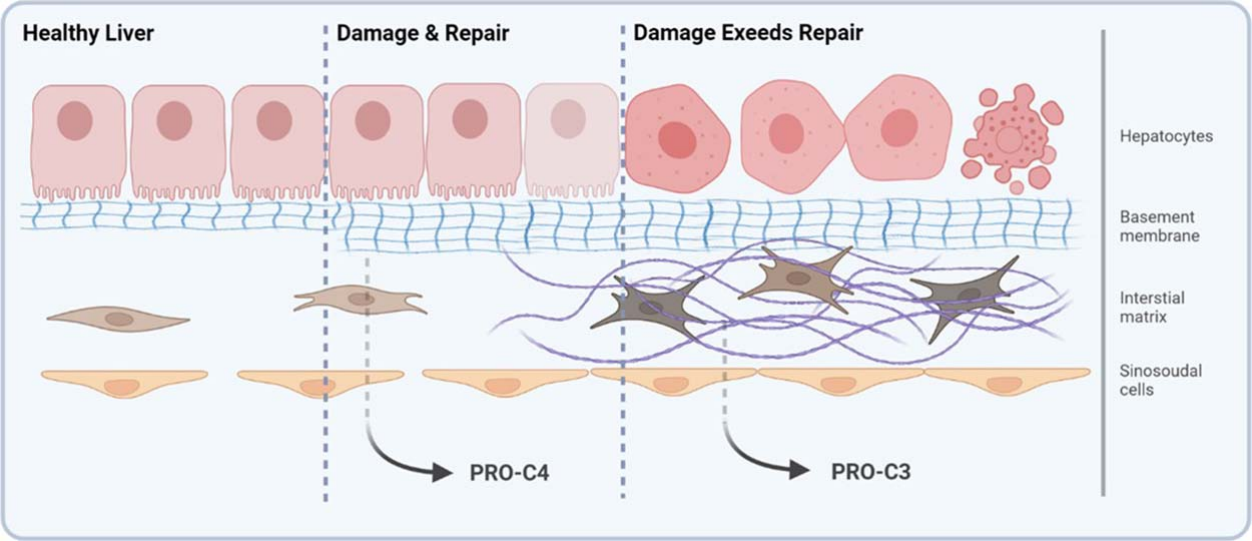
Schematic illustration of the localization of the basement membrane and the interstitial extracellular matrix (ECM) in the liver. Hepatocytes are embedded by the basement membrane ECM, which mainly consist of the networking collagen, type IV, and laminins. While allowing for both tissue maintenance and recovery, the basement membrane also facilitates and controls the interaction between intracellular and extracellular environments. The basement membrane is as such the first line of defense consequent to hepatocyte injury. The interstitial ECM is produced by activated hepatic stellate cells active in response to continued injury and mainly consists of the dense impermeable fibrillar collagens, type I and III collagens.

Damage to the endothelium results in fibroblast activation and perpetuates fibroblast activity in the extracellular matrix (ECM) of the liver. The ECM can be divided into the basement membrane (BM) and interstitial matrix. The liver BM lining the hepatocytes is crucial for hepatocyte organization and function and is the first line of defense and repair after injury, as schematically illustrated in Figure 1.^14–16^ While type IV collagen is the main protein of the BM, type III collagen is one of the dominating fibrillar collagens of the fibrotic interstitial ECM, produced by fibroblasts (Figure 1).^17,18^ The 2 ECM compartments can be assessed by measuring biomarkers of formation of type III (PRO-C3) and type IV (PRO-C4) collagen.^19^ PRO-C3 specifically measures the N-terminal propeptide of type III collagen released by a disintegrin and metalloproteinase with thrombospondin motifs 2 (ADAMTS-2) and, thus, formation of type III collagen. PRO-C4 measures the internal epitope in the 7S domain of type IV collagen, quantifying the BM.^20–22^

The aim of the current study was to investigate fibrosing endotypes at baseline and following weight loss in patients with advanced NAFLD. We hypothesized that markers of fibrogenesis within the BM and the interstitial matrix may define different treatment amendable endotypes amenable to identification with noninvasive tests. This information could be used for patient endotyping at baseline in clinical studies and to support future implementation of personalized therapeutic strategies.

## METHODS

### Participants and intervention

All research was conducted in accordance with both the Declarations of Helsinki and Istanbul. All research was approved by the appropriate ethics institutional review committee. Patients with clinically confirmed NAFLD, who were weight stable (±3%) since diagnosis, were recruited from hepatology clinics at a single tertiary center (The Newcastle upon Tyne Hospitals NHS Foundation Trust) in Newcastle upon Tyne, UK. Patients were prescribed an 8- to 12-week VLCD intervention (∼800 kcal/d) using meal replacement products (Optifast, Nestlè Health Science; nutritional content: fat 19.4% kcal; carbohydrate 43.4% kcal; fiber 3.5% kcal; and protein 33.7% kcal).^23^ The study protocol was approved by North East-Newcastle & North Tyneside 1 Research Ethics Committee (REC reference: 18/NE/0179) (ISRCTN Register: ISRCTN85177264). All participants provided written informed consent. There are no objective primary endpoint or response criteria defined. Fasting blood samples were collected and serum generated at baseline and every second week until the end of the VLCD and again 4 weeks post-VLCD and at 9-month follow-up from baseline. Samples were store at −80 °C until analysis. Weight was monitored by an electronic stadiometer (SECA 799, SECA, UK), and liver stiffness and measured after an 8-hour fast using vibration-controlled transient elastography using a FibroScan Mini 430 (Echosens, Paris, France).

### Biomarker measurements

The Protein Fingerprint biomarkers PRO-C3 and PRO-C4 were assessed according to the manufacturer in serum samples from each patient using ELISA (Nordic Bioscience, Herlev, Denmark). Briefly, 96-well streptavidin–coated plates (Roche Diagnostics, Mannheim, Germany) were incubated for 30 minutes at 20 °C with a biotinylated synthetic peptide. A standard peptide for the calibration curve or prediluted sample was added to appropriate wells, followed by peroxidase-conjugated specific monoclonal antibody and incubated for 1 hour at 20°C or overnight 4°C. All the above incubation steps were performed in darkness and included shaking at 300 rpm. After each incubation step, the plate was washed 5 times in washing buffer (20 mM Tris, 50 mM NaCl, pH 7.2). Finally, tetramethylbenzinidine (cat.438OH, Kem-En-Tec Diagnostics, Taastrup, Denmark) was added and incubated for 15 minutes at 20°C. The tetramethylbenzinidine reaction was stopped by adding 0.18 M H_2_SO_4_ as a stopping solution and measured at 450 nm with 650 nm as a reference. A calibration curve was plotted using a 4-parametric mathematical fit model.

To assess differences in fibrosing response in relation to weight loss, biomarker levels were divided into high and low categories defined as above or below the median at baseline. If baseline levels were the same as the defined cutoff, the patient was included in the low group. The cutoffs were 11.4 ng/mL for PRO-C3 and 263.5 ng/mL for PRO-C4.

### Statistical analysis

**FIGURE 2:**
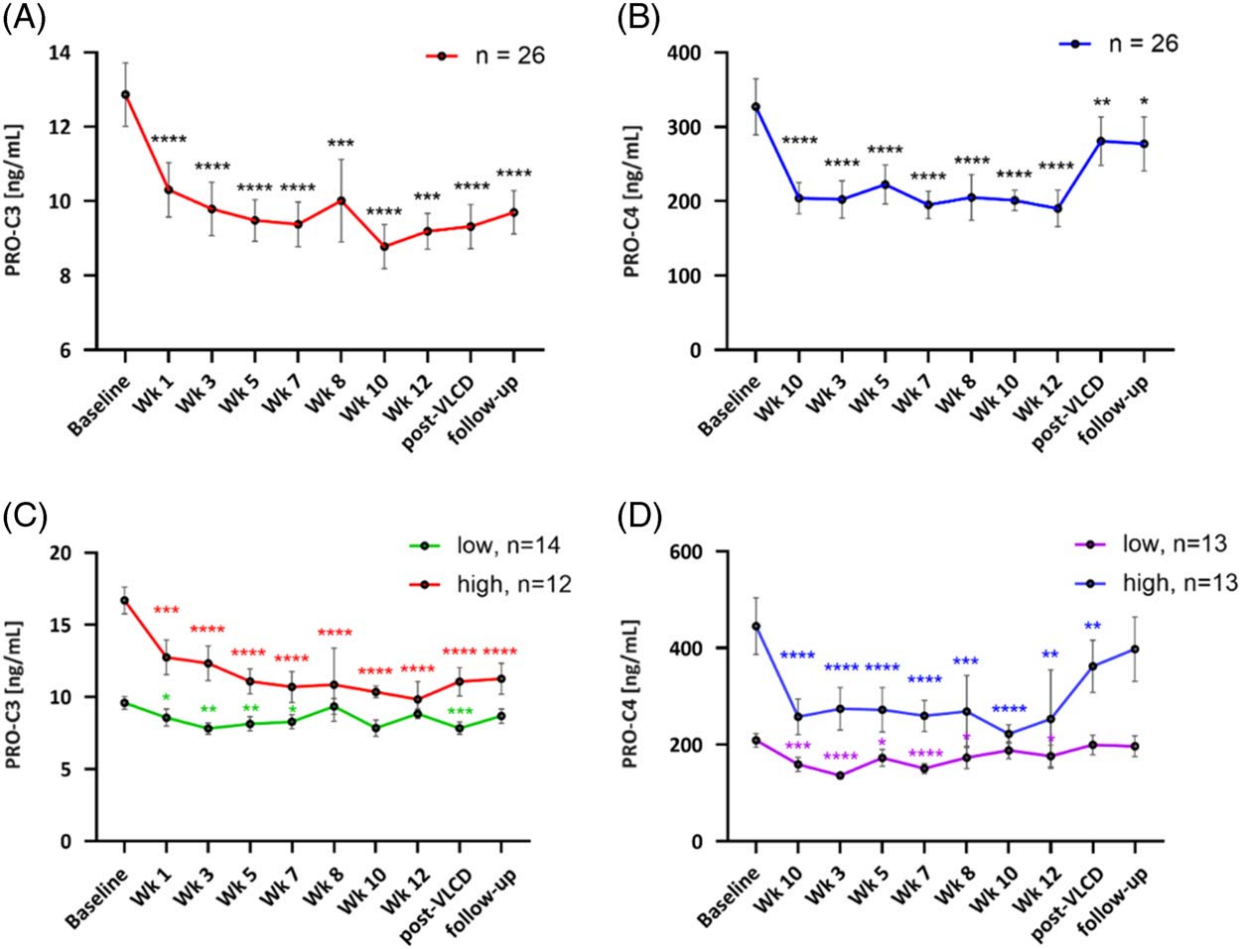
Biomarker trajectory in patients following successful weight loss. (A) PRO-C3 (ng/mL) and (B) PRO-C4 (ng/mL) were both biomarkers in all patients from baseline to end of VLCD (week 12) and 4 weeks post-VLCD and at 9-month follow-up. (C) PRO-C3 (ng/mL) and (D) PRO-C4 (ng/mL) both biomarkers separated into high and low levels at baseline, separated by the median, and followed throughout the course of the study, that is, baseline to week 12, post-VLCD, and 9-month follow-up. Estimated mean values (based on linear mixed model). Statistical difference from baseline to all time points is assessed and indicated as * is *p* < 0.05, ** if *p* < 0.01, *** if *p* < 0.001, and **** if *p* < 0.0001. Abbreviation: VLCD, very low-calorie diet.

A Mann-Whitney test was used to compare patient clinical characteristics at baseline to the end of diet. Similarly, the difference in clinical parameters between high and low groups of biomarkers at baseline was assessed using the Mann-Whitney test. To assess the change in biomarker levels at all timepoints (Figure 2A, B), mean values were estimated (based on a linear mixed model) to take paired data into account. Missing values are implicitly imputed in this way. The relationship between mean values across all timepoints was assessed as a paired *t* test. Graphs and statistical analyses were performed using MedCalc version 19.3 (MedCalc Software Ltd, Ostend, Belgium) or R version 4.1.3 (R Core Team, Vienna, Austria) and GraphPad Prism version 8 (GraphPad Software, La Jolla, CA). Data are shown as median and interquartile range. A *p*-value of *p* < 0.05 was considered statistically significant and indicated as * is *p* > 0.05, ** if *p* > 0.01, *** if *p* > 0.001, and **** if *p* > 0.0001.

## RESULTS

### Improved metabolic and liver profiles were observed following VLCD-induced weight loss

Clinical characteristics are presented in Table 1 and include both baseline and post-VLCD demographics and biomarker parameters. The patients’ characteristics are comparable to that of other NALFD studies, albeit with a slightly higher BMI.^24–26^ Body mass index, aspartate aminotransferase, alanine aminotransferase, liver stiffness by vibration-controlled transient elastography, fasting glucose and gamma-glutamyl transferase were all significantly lowered by the weight loss VLCD (*p* = 0.019–0.0001), as expected as observed in comparable weight loss studies.^27^ PRO-C3 was significantly decreased post-VLCD in contrast to baseline; however, PRO-C4 was not.

**TABLE 1:** Patient characteristics

Biomarker	Baseline	Post-VLCD	*p*
Age (y)	55.5 (50.0, 60.8)	—	—
Sex (n) male/female	16/10	—	—
BMI (kg/m^2^)	39.7 (36.5, 46.0)	34.7 (31.8, 40.9)	<0.0001
Body fat (%)	44.4 (40.4, 49.5)	—	—
Liver stiffness (kPa)	10.0 (8.0, 14.0)	7.2 (5.9, 8.8)	0.0092
Total cholesterol (mmol/L)	4.2 (3.8, 5.1)	4.7 (3.9, 5.0)	0.5730
Triglycerides (mmol/L)	1.6 (1.2, 2.5)	1.4 (1.0, 2.2)	0.0031
HDL (mmol/L)	1.0 (0.9, 1.4)	1.1 (1.0, 1.4)	0.6930
AST (IU/L)	28.5 (21.2, 46.5)	—	—
ALT (IU/L)	40.0 (26.5, 59.2)	—	—
HA (ng/mL)	42.0 (34.3, 107.7)	55.5 (31.9, 83.7)	0.2140
GGT (IU/L)	69.0 (31.0, 93.0)	32.0 (24.0, 42.5)	0.0002
Fasting glucose (mmol/L)	7.2 (5.8, 9.5)	5.4 (5.1, 5.8)	0.0001
HbA1c (mmol/mol)	48.0 (40.0, 60.5)	—	—
Insulin (pmol/L)	102.0 (74.8, 173.0)	—	—
TIMP-1 (ng/mL)	267.5 (216.3, 301.4)	247.8 (203.4, 279.1)	<0.0001
PRO-C3 (ng/mL)	11.4 (10.1, 16.0)	8.9 (7.2, 10.7)	<0.0001
PRO-C4 (ng/mL)	263.5 (236.9, 356.3)	230.4 (183.4, 298.4)	0.0183

*Note:* The *p*-values are from paired *t* tests.

Abbreviations: ALT, alanine aminotransferase; AST, aspartate aminotransferase; BMI, body mass index; GGT, gamma-glutamyl transferase; HA, hyaluronic acid; HbA1c, hemoglobin A1C; VLCD, very low-calorie diet.

Serum PRO-C3 and PRO-C4 dynamics over time are shown in Figure 2A, B. PRO-C3 and PRO-C4 were significantly reduced by the weight loss from week 1 until the end of VLCD (*p* < 0.001). Post-VLCD, PRO-C3 did not return to baseline level. PRO-C4 increased toward baseline levels post-VLCD.

Patient stratification into “high-risk, high-fibrogenesis” and “low-risk, low-fibrogenesis” endotypes based on baseline PRO-C3 and PRO-C4 measurements was performed (PRO-C3 > or ≤ 11.4 ng/mL and PRO-C4 > or ≤ 263.5 ng/mL). Data revealed different biomarker trajectories following completion of the VLCD and at follow-up between the 2 groups shown in Figure 2C, D. Patients in the high-fibrogenesis group (baseline PRO-C3 11.4 ng/mL) exhibited significant PRO-C3 reductions (*p* < 0.0001) at week 12 compared to baseline, while the low-fibrogenesis group did not exhibit a significant reduction. In addition, at post-VLCD and at 9 month follow-up, PRO-C3 was not found to increase again in either group of patients. Biomarker values are shown at all timepoints. Considering PRO-C4, the high-fibrogenesis group (baseline PRO-C4 > 263.5 ng/mL) also demonstrated significant reductions from baseline to week 12 (*p* < 0.01). However, interestingly, the levels were found to start increasing after the end of the VLCD with no significant difference between baseline and follow-up, that is, 6 months after patients returned to a normal diet.

### Clinical parameters in fast or slow progressive endotypes based on PRO-C3 and PRO-C4

In Tables 2 and 3, patient data are separated into change over time in all comers, and high or low PRO-C3 and PRO-C4 strata based on median levels at baseline. Insulin levels were found to be significantly different (*p* < 0.0001 at baseline and *p* < 0.05 at week 12) between PRO-C3 strata at baseline and end of VLCD. No other clinical parameters were found to differ between the fibrogenesis biomarker endotypes. Interestingly, in Table 3 in allcomers, HDL, total cholesterol and triglycerides did not change over time, and more importantly other biomarkers did not change significantly when stratified according to biomarker levels, as shown in Table 4, suggesting that standard biomarkers such as alanine aminotransferase and aspartate aminotransferase provide a different value and information as compared to fibrogenesis biomarkers.

**TABLE 2:** Patient characteristics baseline, stratified in high and low biomarker levels

Biomarker	Low PRO-C3	High PRO-C3	*p*	Low PRO-C4	High PRO-C4	*p*
BMI (kg/m^2^)	38.9 (36.6, 42.2)	40.5 (36.0, 47.1)	0.6130	38.3 (36.0, 44.5)	40.3 (37.4, 45.4)	0.6860
ALT (IU/L)	40.5 (32.8, 57.8)	36.0 (26.5, 69.2)	0.6630	28.0 (26.0, 61.0)	42.0 (29.5, 58.5)	0.6440
AST (IU/L)	25.0 (20.2, 35.0)	35.0 (22.2, 53.5)	0.1760	26.0 (24.0, 50.0)	31.0 (20.0, 42.5)	0.5250
HA (ng/mL)	40.9 (33.4, 130.9)	43.5 (34.8, 84.6)	0.9900	89.8 (41.1, 149.7)	40.9 (29.9, 75.4)	0.1560
HbA1c (mmol/mol)	48.0 (43.8, 61.8)	49.0 (39.2, 58.2)	0.8240	51.0 (38.5, 57.0)	48.0 (42.5, 62.5)	0.7280
GGT (IU/L)	70.0 (29.0, 92.0)	65.5 (35.5, 89.0)	0.8610	37.0 (26.0, 99.0)	69.5 (38.5, 87.5)	0.5650
Total cholesterol (mmol/L)	4.2 (4.1, 5.0)	4.3 (3.5, 5.1)	0.5090	3.6 (3.4, 4.6)	4.4 (4.0, 5.0)	0.1330
Triglycerides (mmol/L)	1.4 (1.3, 2.0)	1.8 (1.2, 2.9)	0.1530	2.1 (1.3, 2.9)	1.5 (1.2, 2.3)	0.4870
HDL (mmol/L)	1.4 (1.1, 1.4)	1.0 (0.9, 1.1)	0.0433	1.0 (0.9, 1.0)	1.2 (0.9, 1.4)	0.2090
Fasting glucose (mmol/L)	6.4 (5.5, 8.4)	7.6 (6.2, 9.5)	0.2470	7.0 (6.2, 8.6)	7.3 (5.7, 9.3)	0.9310
Insulin (pmol/L)	74.8 (62.0, 82.2)	155.5 (118.8, 223.5)	0.0012	91.0 (77.6, 119.5)	109.5 (76.3, 175.2)	0.7900
ELF	9.3 (9.0, 10.1)	9.5 (9.3, 10.3)	0.2230	9.9 (9.4, 10.6)	9.3 (9.1, 10.0)	0.2720

*Note:* Patient characteristics in patients stratified into high/low fibrogenesis based on baseline type III and type IV collagen fibrogenesis markers.

*p*-values indicate a significant difference from the high biomarker group, calculated using The *p*-values are from 2-sample *t* tests.

Abbreviations: ALT, alanine aminotransferase; AST, aspartate aminotransferase; BMI, body mass index; ELF, Enhanced Liver Fibrosis; GGT, gamma-glutamyl transferase; HA, hyaluronic acid; HbA1c, hemoglobin A1C.

**TABLE 3:** Patient characteristics at baseline and EOT

Biomarker	Baseline	EOT	*p*
BMI (kg/m^2^)	39.7 (36.5, 46.0)	33.0 (31.0, 35.0)	<0.0001
Total cholesterol (mmol/L)	4.2 (3.8, 5.1)	4.2 (3.7, 5.0)	0.1510
Triglycerides (mmol/L)	1.6 (1.2, 2.5)	1.4 (1.0, 1.9)	0.1640
HDL (mmol/L)	1.0 (0.9, 1.4)	1.1 (0.9, 1.5)	0.7000
AST (IU/L)	28.5 (21.2, 46.5)	22.0 (20.0, 27.5)	0.0022
ALT (IU/L)	40.0 (26.5, 59.2)	25.0 (21.0, 33.0)	0.0023
HA (ng/mL)	42.0 (34.3, 107.7)	38.0 (27.9, 109.0)	0.0247
GGT (IU/L)	69.0 (31.0, 93.0)	38.0 (23.0, 48.0)	0.0004
HbA1c (mmol/mol)	48.0 (40.0, 60.5)	39.0 (38.0, 46.0)	<0.0001
Insulin (pmol/L)	102.0 (74.8, 173.0)	44.2 (33.6, 96.7)	0.0091
PRO-C3 (ng/mL)	11.4 (10.1, 16.0)	9.3 (8.5, 10.4)	0.0010
PRO-C4 (ng/mL)	263.5 (236.9, 356.3)	184.6 (153.2, 247.2)	0.0064

*Note:* The *p*-values are from paired *t* tests.

Abbreviations: ALT, alanine aminotransferase; AST, aspartate aminotransferase; BMI, body mass index; EOT, End Of Treatment; GGT, gamma-glutamyl transferase; HA, hyaluronic acid; HbA1c, hemoglobin A1C.

**TABLE 4:** Change from BL to EOT, stratified in high and low biomarker levels, and absolute change

Biomarker	Low PRO-C3	High PRO-C3	*p*	Low PRO-C4	High PRO-C4	*p*
BMI (kg/m^2^)	−4.7 (−5.4, −3.2)	−6.6 (−7.0, −4.7)	0.2230	−6.4 (−7.1, −5.0)	−4.8 (−5.6, −3.0)	0.1430
ALT (IU/L)	−5.5 (−21.2, 1.8)	−9.0 (−29.0, −1.0)	0.4210	−6.0 (−38.5, −4.0)	−7.5 (−21.2, 1.2)	0.3160
AST (IU/L)	−3.0 (−16.0, 0.5)	−6.5 (−26.5, −2.8)	0.2910	−5.0 (−27.5, −4.0)	−3.5 (−16.2, 1.0)	0.3320
HA (ng/mL)	−7.1 (−28.1, 2.4)	−23.7 (−44.5, −6.7)	0.5050	−24.1 (−35.6, 0.9)	−11.0 (−24.4, 2.0)	1.0000
HbA1c (mmol/mol)	−5.5 (−9.0, −1.2)	−8.0 (−13.5, −1.5)	0.3990	−9.0 (−13.0, −2.0)	−8.0 (−10.2, −1.0)	0.5610
GGT (IU/L)	−12.0 (−28.0, 5.0)	−12.0 (−47.5, −4.5)	0.2990	−11.0 (−57.0, −2.0)	−12.0 (−34.0, −4.0)	1.0000
Total cholesterol (mmol/L)	−0.3 (−0.6, 0.1)	−0.3 (−0.6, 0.4)	0.5610	−0.3 (−0.6, 0.3)	−0.3 (−0.6, 0.1)	0.9580
Triglycerides (mmol/L)	−0.2 (−0.6, −0.1)	0.1 (−0.5, 0.3)	0.6320	−0.6 (−1.2, 0.4)	−0.2 (−0.5, 0.2)	0.5950
HDL (mmol/L)	0.0 (−0.1, 0.1)	0.0 (−0.1, 0.1)	0.2960	−0.0 (−0.1, 0.0)	0.0 (−0.1, 0.1)	0.4840
Insulin (pmol/L)	−24.8 (−45.2, −11.4)	−62.8 (−130.9, 0.4)	0.2900	−63.4 (−93.7, −22.6)	−28.3 (−48.9, 8.9)	0.2980
ELF	−0.4 (−0.8, −0.1)	−0.8 (−1.1, −0.3)	0.3630	−0.3 (−0.9, −0.1)	−0.7 (−1.0, −0.3)	0.5290

*Note:* The *p*-values are from 2-sample *t* tests.

Abbreviations: ALT, alanine aminotransferase; AST, aspartate aminotransferase; BMI, body mass index; ELF, Enhanced Liver Fibrosis; EOT, End Of Treatment; GGT, gamma-glutamyl transferase; HA, hyaluronic acid; HbA1c, hemoglobin A1C.

## DISCUSSION

The high prevalence of NAFLD in the general population, combined with substantial interpatient variation in disease severity and natural history,^28^ highlights the pressing need to better understand patient heterogeneity and identify predictive endotypes and so triage patients toward more personalized therapeutic strategies.^8^ This is a pressing need as ill-defined patient heterogeneity also hampers the development of effective pharmacological therapies. Precise identification of patient endotypes more susceptible to treatment may improve patient care, assist in targeted drug development, and, thus, help realize personalized medicine in NAFLD.

In NASH studies, ~25% of patients exhibit progressive fibrosis, 25% spontaneously reverse fibrosis, and leaving 50% with stable fibrosis,^29^ albeit this conclusion is heavily influenced by the inaccuracy of the liver biopsy.^30,31^ There is a need to better understand and implement NITs for assessing fibrogenesis.^32^ Type III collagen formation is becoming increasingly recognized as a biomarker for liver fibrosis activity, in which several studies have shown high levels of PRO-C3 to be associated with fibrosis progression and worse outcomes, as well as increased treatment response in subjects with higher levels of PRO-C3.^9,12,33^

Relevant experience in the identification of endotypes may be gained from other disease areas, such as osteoporosis, where there are already accepted treatments and FDA-validated biomarkers. The tissue balance, the ratio between tissue formation and tissue degradation^13^ has been used extensively in the osteoporosis field to document potential efficacy of given treatments. For example, antiresorptive agents dose-dependently inhibited bone resorption as quantified by CTX-I, whereas anabolic interventions dose-dependently stimulated bone formation quantified by type I collagen formation.^13^ Both treatment regimens resulted in increased bone mineral density, bone mass, and a reduction of fractures (outcome).^34^ Clearly, anticatabolic or anabolic treatments were developed with a different set of biomarkers. In direct alignment, in the osteoarthritis field, a low repair and cartilage formation endotype identified by type II collagen formation (PRO-C2)^35^ in serum responded significantly better to anabolic treatment (FGF-18 used intra-articulacy).^36^ This endotype was associated with faster progression of joint damage, as quantified by MRI. Thus, collagen formation and degradation biomarkers have, in several therapeutic areas, been shown to assist in drug development.

With respect to the effect of weight loss on remodeling on the interstitial ECM, the standard fibrotic ECM, we saw a clear reduction in the fibrogenesis biomarker PRO-C3 and the BM formation biomarkers PRO-C4 but not in HDL, total cholesterol, and triglycerides. This attenuation of fibrosis activity rebalanced many patients into the healthy reference range. Interesting, as active fibroblasts produce PRO-C3, it appears that the proposed deactivation of fibroblasts is more rapid than their reactivation as PRO-C3 was not observed to rise again following completion of the VLCD intervention. This may have interesting implications for the long-term clinical benefits of weight loss or even fasting.

With respect to the BM, the type IV collagen formation levels were found to increase again following completion of the VLCD, and as such, exhibit a different dynamic trajectory compared to PRO-C3. This reflects the differences in the tissue turnover of the intestinal ECM and the BM, and suggests different turnover mechanisms of each individual compartment. Further research is needed to fully understand the mechanisms, with respect to damage and repair, and the transition into fibrosis progression from a continuous insult of the basement into fibrosis of the interstitial matrix membrane versus regression.^37^ In direct alignment, the fibrosis histopathology in early disease (fibrosis stage F1-2) mostly is happening in the BM.^18^ We are beginning to understand that fibrosis biomarkers may be modulated in the early fibrosis profile, and that is somewhat different from late-stage disease. As such, remodeling of the BM is found to be more prominently affected following weight loss, indicating altered tissue balance, which may support and promote liver repair.^15,38^ Of note, it was not the same patients who had elevated levels of both type III and IV collagen formation. Further research is needed to investigate the trajectories of how the levels of these biomarkers interchange and relate to insults and repair.

A key take-home message is that in fibrosing diseases such as NAFLD, characterizing tissue balance and fibrosis remodeling may be of importance, targeting different interventions to different endotypes. Being achievable and evident within a short timeframe, fibrogenesis activity rather than the absolute stage could be an important endpoint for treatment. Active fibroblasts are the key cellular components of fibrosis development, producing an interstitial matrix and mainly consisting of the fibrillar collagens. Active fibroblasts produce type III collagen,^39^ which may be quantified in the serum as pro-peptides of type III collagen. Thus, for antifibrinogenesis treatments to provide efficacy, the endotype with high fibrosis cellular activity may be important to identify. In the current study, patients with high levels of type III collagen fibrogenesis responded rapidly and significantly to different dietary interventions compared to patients with a low level of PRO-C3 at baseline. A few other *post hoc* analysis studies have shown that patients with high levels of PRO-C3, patients with active fibrogenesis, have increased levels of progression of fibrosis and liver related outcomes.^9,40,41^

Of interest, there was a high association between levels of insulin and PRO-C3 at baseline and at follow-up. This is in line with previous observations of a connection between insulin and PRO-C3,^42^ and may be related to the fact that insulin is upregulated in insulin resistance in nontreated people with prediabetes,^43^ which is in accordance with type 2 diabetes being a major risk factor for progression of NAFLD. Of particular interest is the large difference in change in HbA1c in the PRO-C3 high versus PRO-C3 low group after the intervention period. While the groups at baseline had similar levels, the decrease in the high group was 10 mmol/mol (*p* < 0.001) versus only 3.5 mmol/mol (*p* < 0.001) in the low group, although the difference between groups only reached borderline significance. This may suggest that a high fibrotic endotype is associated with a better glucose response to intervention. A similar phenomenon has previously been reported for another fibroblast collagen formation biomarker, PRO-C6, in relation to response to the PPAR therapy. Those patients with the highest PRO-C6 levels had a significantly larger effect size^44^ on HbA1c, in alignment with the present findings. This potential fibrotic endotype and response to intervention on glucose deserves additional attention.

The limitation of the current study is the low number of patients; thus, data need to be reproduced in other studies. In addition, liver fibrosis assessments were challenged by a high coefficient of variation (CV) on liver stiffness measurements, and no other liver fibrosis validation was assessable.

In conclusion, the fibrosis field may benefit from an understanding of NAFLD endotypes at baseline to improve intervention. A “high-risk, high-fibrogenesis” patient endotype may identify patients that respond better to treatments and interventions that read through to affect active fibroblasts, whether directly targeting these cells or, as in the case of weight loss, targeting upstream mechanisms. Similarly, there is a need to identify the low fibrosis resolution endotypes and align biomarkers and treatments according to their mechanisms of action as is done in other fields
